# miRNA Expression Profiling in Migrating Glioblastoma Cells: Regulation of Cell Migration and Invasion by miR-23b via Targeting of Pyk2

**DOI:** 10.1371/journal.pone.0039818

**Published:** 2012-06-22

**Authors:** Joseph C. Loftus, Julianna T. D. Ross, Kimberly M. Paquette, Vincent M. Paulino, Sara Nasser, Zhongbo Yang, Jean Kloss, Seungchan Kim, Michael E. Berens, Nhan L. Tran

**Affiliations:** 1 Department of Biochemistry and Molecular Biology, Mayo Clinic Arizona, Scottsdale, Arizona, United States of America; 2 Cancer and Cell Biology Division, The Translational Genomics Research Institute, Phoenix, Arizona, United States of America; 3 Computational Biology Division, The Translational Genomics Research Institute, Phoenix, Arizona, United States of America; University of Edinburgh, United Kingdom

## Abstract

**Background:**

Glioblastoma (GB) is the most common and lethal type of primary brain tumor. Clinical outcome remains poor and is essentially palliative due to the highly invasive nature of the disease. A more thorough understanding of the molecular mechanisms that drive glioma invasion is required to limit dispersion of malignant glioma cells.

**Methodology/Principal Findings:**

We investigated the potential role of differential expression of microRNAs (miRNA) in glioma invasion by comparing the matched large-scale, genome-wide miRNA expression profiles of migrating and migration-restricted human glioma cells. Migratory and migration-restricted cell populations from seven glioma cell lines were isolated and profiled for miRNA expression. Statistical analyses revealed a set of miRNAs common to all seven glioma cell lines that were significantly down regulated in the migrating cell population relative to cells in the migration-restricted population. Among the down-regulated miRNAs, miR-23b has been reported to target potential drivers of cell migration and invasion in other cell types. Over-expression of miR-23b significantly inhibited glioma cell migration and invasion. A bioinformatics search revealed a conserved target site within the 3′ untranslated region (UTR) of Pyk2, a non-receptor tyrosine kinase previously implicated in the regulation of glioma cell migration and invasion. Increased expression of miR-23b reduced the protein expression level of Pyk2 in glioma cells but did not significantly alter the protein expression level of the related focal adhesion kinase FAK. Expression of Pyk2 via a transcript variant missing the 3′UTR in miR-23b-expressing cells partially rescued cell migration, whereas expression of Pyk2 via a transcript containing an intact 3′UTR failed to rescue cell migration.

**Conclusions/Significance:**

Reduced expression of miR-23b enhances glioma cell migration *in vitro* and invasion *ex vivo* via modulation of Pyk2 protein expression. The data suggest that specific miRNAs may regulate glioma migration and invasion to influence the progression of this disease.

## Introduction

Glioblastoma (GB) is the most common adult brain tumor and is characterized by its extensive infiltration into normal brain tissue. This aggressive invasion effectively precludes complete surgical resection and all but assures recurrent tumor growth. Moreover, invading glioma cells reduce transcription of proapoptotic and proliferation genes [1] concordant with decreased susceptibility to cytotoxic agents [2], [3] providing them with an additional mechanism for resisting current radiological and chemotherapeutic treatment regimens. Malignant cells can invade over a significant distance in the brain parenchyma, commonly along extracellular matrices of blood vessels and nerve fiber tracts to initiate additional tumor growth. Notably, this invasive characteristic is not shared by nonglial cells that metastasize to the brain from other primary tumor sites highlighting the unique biology of invasive glioma cells. This behavior carries with it a very poor clinical prognosis. The median survival of GB patients following diagnosis is 15 months with an overall five-year survival of just under 10% [4]. Therefore, a more thorough understanding of the molecular mechanisms that drive glioma invasion is required to develop more effective therapeutic treatment [5].

Global gene expression profiling of glioblastoma has provided substantial insights into genetic alterations in GB and fundamental signaling pathways [6], [7], [8]. Importantly, these genetic alterations have helped establish a molecular classification of GB into distinct subclasses with important clinical consequences associated with response to current treatment regimens [9], [10]. Less well characterized is the specific set of genes that serve as drivers of the invasive phenotype of GB. In previous studies, we investigated the transcriptome of migratory glioma cells *in vitro* [1] and from invasive cells *in situ* [11], [12] to identify a set of genes differentially expressed in invading glioma cells. Comprehensive analysis of the transcriptome profiles of matched populations of migratory and stationary glioma cells produced an invasion signature that was validated at the transcriptional and translational levels in clinical samples [13]. Functional validation of these genes in glioma migration and invasion suggests potential unique targets to specifically inhibit the invasive glioma cell population.

The molecular mechanisms initiating or regulating the expression of potential drivers of glioma invasion remain very poorly defined. MicroRNAs (miRNAs) are a class of endogenous, small, non-protein coding RNA molecules that serve as post-transcriptional regulators of gene expression. More than 1200 human miRNAs have been identified and many play important roles in diverse biological processes, including cellular proliferation, cell motility, cell cycle determination, differentiation, apoptosis, neuronal patterning, and development [14], [15]. Alterations in specific miRNA expression levels have been identified in a number of cancers where they may function to either inhibit the expression of tumor suppressor genes or by allowing increased expression of oncogenes [15]. Several studies have identified various miRNAs that have potential functional importance in GB [16], [17], [18]. miR-21 is overexpressed in GB and is linked to increased tumor growth through an inhibition of apoptosis [19]. Similarly, increased expression of miR-221 in GB is associated with aberrant cell cycle progression and increased proliferation [20], [21]. miRNA expression profiling has also demonstrated reduced expression of several miRNAs in GB including miR-7, miR-128, miR124, miR137, and miR-218 which have been linked to alterations in proliferation, differentiation, invasion, and stem cell self-renewal [18], [22], [23].

To investigate the regulatory mechanisms governing the invasive signature and to prioritize our investigation of potential gene candidates regulating glioma cell migration and invasion, we analyzed miRNA expression profiles in matched populations of migrating cells versus migration-restricted cells in seven well-established glioma cell lines. Data analysis revealed 72 consistently differentially expressed miRNAs that exhibited similar expression patterns in all seven glioma cell lines with a smaller conserved set of miRNAs that were down regulated in the migrating cell population. Among the miRNAs significantly down regulated in the invasive cell population *in vitro* was miR-23b which was also down regulated in the invasive edge *in situ* in clinical GB specimens. One of the predicted targets for miR-23b is the focal adhesion kinase Pyk2 that we have previously identified as an important regulator of glioma cell migration [24], [25]. In this report, we confirm that Pyk2 is targeted by miR-23b and demonstrate that increased expression of miR-23b inhibits glioma cell migration *in vitro* and invasion *ex vivo* while knockdown of miR-23b stimulates glioma cell migration. Increased expression of miR-23b reduced Pyk2 expression suggesting that reduced expression of miR-23b facilitates the enhancement of glioma cell migration and invasion via modulation of Pyk2 expression. Identification of miRNAs associated with glioma migration and defining the mechanistic basis of their modulation of the invasive phenotype may provide insights into potential targets for novel therapeutic strategies aimed at limiting glioma invasion and improved clinical outcomes.

## Materials and Methods

### Cell culture

Human glioblastoma cell lines A172, T98G, U87, SNB19, U251 (American Type Culture Collection), SF767 (University of California at San Francisco), G112MS [26] and 293T packaging cells were maintained in Dulbecco's modified Eagle's medium (DMEM), supplemented with 10% fetal calf serum in a 37°C, 5% CO_2_ atmosphere at constant humidity.

### Reagents, antibodies, and immunoblot analysis

Human placental laminin, the anti-FLAG M2 monoclonal antibody, and anti-actin antibody were obtained from Sigma (St. Louis, MO). Affinity purified polyclonal anti-Pyk2 and anti-FAK antibodies were obtained from Upstate Biotechnology (Lake Placid, NY). Immunoblotting of cellular lysates was performed as previously described [27].

### Radial cell migration assay and isolation of migratory and stationary cell populations

The radial cell migration assay was performed as previously described [28], [29]. Briefly, three thousand cells were seeded as a defined, confluent circular monolayer using a cell sedimentation manifold (CSM, Inc. Phoenix, AZ) on laminin coated 10-well slides. Sixteen hours after seeding the manifold was removed and the cells were allowed to migrate for 24 hours. The migration rates were estimated from photomicrographs (Axiovert, Carl Zeiss Inc., NY) of the diameter of the cell colony taken at the time of manifold removal (time point 0 hours) and at time point 24 hours using image analysis software (Scion Image, Frederick, MD). Isolation of a GB migratory front (rim) and stationary core was performed as previously described [13]. Stationary core and migratory rim cell populations were harvested under an inverted microscope (Axiovert 100, Carl Zeiss Inc., NY) using a P2 pipette in three independent biological replicates. Thirty individual dispersion assays (three 10-well slides) were collected per cell line and subjected to total RNA, including small RNA, isolation.

In certain experiments, glioma cells were seeded in a 6-well plate at 70% confluence and transfected with 100 nM of anti-miR-23b or anti-miR negative control oligonucleotides (Ambion, Austin, TX). At 16 hours post-transfection, cells were recovered in DMEM +10% FBS and seeded for cell migration assay as described above. Transfection efficiency was confirmed by quantitative PCR 48 hours after transfection.

### Clinical Samples and Histology

Fresh human glioblastoma specimens (WHO Grade IV) were collected from patients following informed written consent at Abbott Northwestern Hospital. Samples were submitted to the study under a protocol approved by the Western Institutional Review Board (protocol #20110995). Samples were obtained from patients who underwent primary therapeutic subtotal or total tumor resection performed under image guidance. All tissue samples were obtained at primary resection, and none of the patients had undergone prior chemotherapy or radiation therapy. The samples were immediately frozen on dry ice and cut into 12-µm sections. Histological diagnosis of tumor core and invasive edge was made by neuropathological review by standard light-microscopic evaluation of the sections stained with hematoxylin and eosin. Tumors cells adjacent to necrotic areas and cortical areas, cells with small, regular nuclei, reactive astrocytes and endothelial and blood cells were avoided. Invading GB cells at the invasive edge were identified by means of their nuclear atypia and heteropyknotic staining, consistent with cells within the tumor core. The targeted areas were collected by microscopic dissection under an inverted microscope and processed immediately for RNA isolation.

### RNA extraction, miRNA microarray profiling, and data processing

Total RNA, including small RNA, was isolated using the mirVana miRNA isolation kit (Ambion, Austin, TX). The RNA quality and quantity were assessed using the NanoDrop 2000 (Thermo Scientific, Waltham, MA). The integrity of RNA was determined using a Bioanalyzer 2100 Nano LabChip kit (Agilent Technologies, Santa Clara, CA). Samples selected for the study contained intact RNA with a RIN≥8.0. One hundred ng total RNA was end-labeled with Cy3-pCp following the manufacturer's recommendations using Agilent’s miRNA Complete Labeling and Hyb Kit (Agilent). Labeled miRNA was hybridized to Agilent’s Human 8x15K miRNA Microarrays (V2) based on Sanger miRbase (release 10.1). Images were captured using an Agilent DNA Microarray Scanner set at default settings for miRNA microarrays. Scanned TIFF images were processed using Feature Extractor v. 10.5.1.1. Further quality control and normalization was performed using GeneSpring GX 11 (Agilent). Signals <1 were set to 1 due to GeneSpring’s analysis in log space, negative values were converted as well. Values were divided by the 75th percentile signal on that array to allow for improved comparison between arrays.

Statistical significance of differentially expressed miRNAs between the rim and core cell populations was determined using a two-tail t-test followed by FDR correction using Storey’s method [30]. Hierarchical clustering, which builds clusters based on the hierarchy of groups, was used to visualize the prominence of miRNAs differentiating rim and core cell populations. Hierarchical clustering of the differentially expressed miRNA was performed using Spearman’s correlation (1-correlation) as distance and complete linkage to group clusters. Clustering and t-test were performed using MATLAB (MathWorks, Natick, MA).

### Quantitative Reverse Transcription-PCR (qRT-PCR) analysis of miRNAs

Complementary DNAs (cDNAs) were synthesized using 25 ng of total RNA following the manufacturer's directions in the TaqMan microRNA Reverse Transcription kit and miRNA-specific stem-loop primers (Life Technologies, Carlsbad, CA). Quantitative PCR analyses of miRNAs and *18s* ribosomal RNA were carried out in triplicate in a 384-well plate using a LightCycler 480 (Roche Applied Sciences, Indianapolis, IN) with TaqMan fluorescence signal detection (Life Technologies) after each cycle of amplification. Crossing points of miRNA vs. *18S* ribosomal RNA were used to calculate relative fold up-/down-regulation in the migrating cells as previously described [13].

### Adenovirus production and infection

The design and performance of a recombinant adenovirus encoding wild-type Pyk2 was previously described [25]. This clone, designated Pyk WT, contains the entire coding sequence and an additional 725 base pairs of the 3' UTR. To construct a recombinant adenovirus encoding Pyk2 devoid of 3' UTR, designated Pyk 3Kb, the Pyk2 coding sequence was amplified by PCR and ligated into the adenoviral transfer vector pShuttle-CMV. E1-deleted adenoviruses were prepared using the Ad-Easy systems as described [31]. Recombinant adenoviruses were propagated in 293 cells, clonally isolated, and titered. Subconfluent cultures of target cells were infected at matched multiplicity of infection (MOI). Immunoblotting of adenovirus infected cells was done with matched cell samples plated on the same substrate as used for the migration assay.

### Lentiviral transduction

The lentiviral vector pMIRNA1 containing a pre-miRNA-23b sequence was obtained from System Biosciences, LLC. (Mountain View, CA). The miRNA-23b precursor construct was packaged into VSV-G pseudotyped lentiviral particles following transfection of 293T cells using the pPACKH1 packaging plasmid mix (System Biosciences) according to the manufacturer's instructions. For lentiviral transduction of target cells, lentiviral-containing supernatants were collected from packaging cells at 48 and 72 hours after transfection, concentrated by PEG precipitation, and added to subconfluent cultures of cells with 8 µg/ml polybrene for 4–6 hours. Forty-eight hours after infection, cells were harvested and green fluorescence protein (GFP) positive cells were collected by mass sorting on a FACSAria flow cytometer (BD Biosciences, San Jose, CA).

### Organotypic brain slice invasion assay

An *ex vivo* invasion assay on mouse brain slices was carried out as previously described [32], [33]. Animals were maintained and brain slices obtained under a research protocol approved by the Institutional Animal Care and Use Committee of St. Joseph's Hospital and Medical Center, Barrow Neurological Institute (protocol #275, Nhan L Tran). Briefly, approximately 1×10^5^ glioma cells stably expressing miR-23b and GFP or empty vector control (GFP only) were gently deposited (0.5-μL transfer volume) onto the bilateral putamen of the 400 µm thick slices of freshly isolated 4–6 week-old murine brains. Glioma cell invasion into the brain slices was quantified using a LSM 5 confocal microscope and depth of invasion (z-axis stacks) was calculated as previously described [34].

### Statistical analysis

Independent sample t-test statistical analyses of invasion and migration assay data was performed with GraphPad Prism 5.0 (GraphPad Software, La Jolla, CA). All tests are two-tailed. *p*<0.05 was considered significant.

## Results

### Genome-wide miRNA expression profiling demonstrates differential expression of miRNAs in paired migratory and migration restricted cell populations

**Figure 1:**
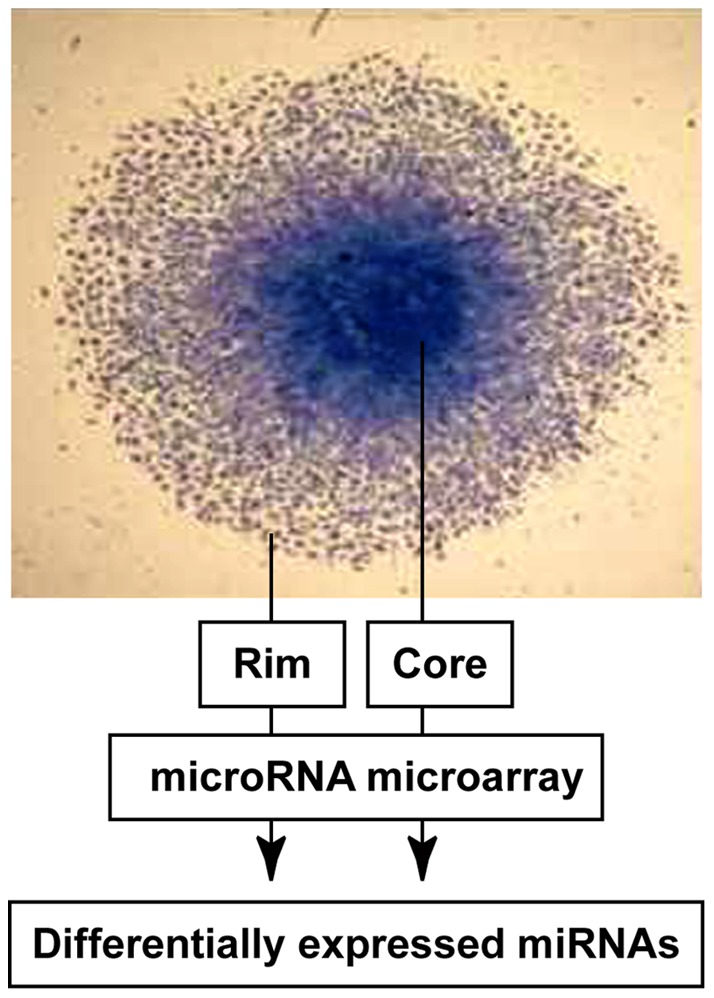
miRNA analysis of migration active and migration restricted glioma cells *in vitro*. Glioma cell lines were seeded onto 10-well glass slides coated with 10 µg/ml laminin and allowed to migrate for 24 hours. Total RNA, including small RNA, was isolated from microdissected migrating (rim) and migration-restricted (core) cell populations. Total RNA (100 ng) was end-labeled with Cy3 and hybridized to Agilent human miRNA microarrays.

**Figure 2:**
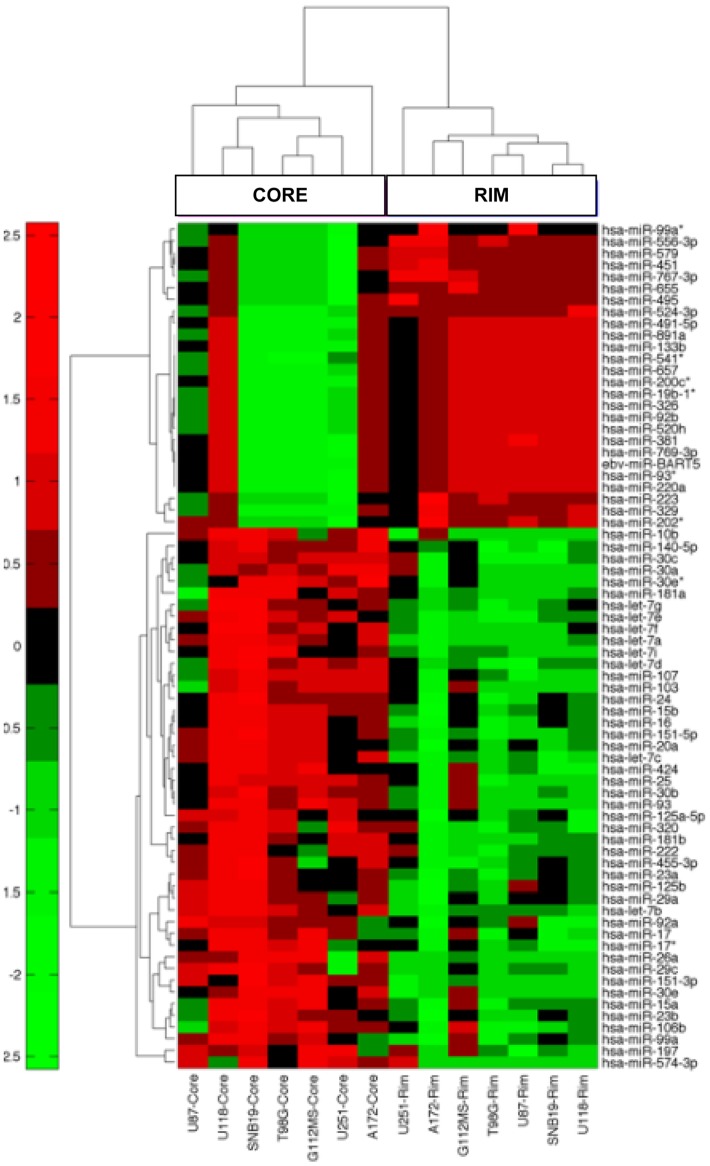
miRNA analysis of glioma cell migration. miRNA expression in paired migration-restricted and migrating cell populations from seven different glioma cell lines was profiled using Agilent human miRNA microarrays. Hierarchical cluster dendrogram of significant differentially expressed miRNAs (*p*<0.01). Red and green color scale represents high and low expression respectively.

To better understand the epigenetic program driving glioma migration and invasion, we profiled global miRNA expression in paired migration-restricted and migrating cell populations using a well-characterized *in vitro* radial migration assay [28], [29] with seven different glioma cell lines. For each cell line, cells were plated as a confluent, circular monolayer then allowed to migrate on laminin for 24 hours. Migration-restricted cells from the core or migrating cells from the rim were harvested and total RNA, including small RNAs, was collected and profiled for miRNA expression using Agilent 8x15K miRNA microarrays (Figure 1). Data analysis revealed a set of 72 miRNAs that were consistently differentially expressed between the core and the rim in all seven glioma cell lines (Table 1 and Table 2). Hierarchical cluster analysis of these miRNAs demonstrate that migrating and migration-restricted cells from a range of glioma cell lines each express a shared subset of miRNAs (Figure 2), suggesting an epigenetic mechanism may help drive invasion of glioma cells *in vivo*.

**Table 1:** Up-regulated miRNAs in migrating glioma cells.

microRNA ID	Fold Change (Rim vs. Core)	*p*-value (T-test)	False Discovery Rate
hsa-miR-99a*	1.99	0.00660	0.02081
hsa-miR-767-3p	1.94	0.00314	0.01455
hsa-miR-202*	1.85	0.00850	0.01999
hsa-miR-556-3p	1.79	0.00414	0.01674
hsa-miR-655	1.78	0.00353	0.01505
hsa-miR-451	1.72	0.00548	0.01963
hsa-miR-495	1.67	0.00949	0.02107
hsa-miR-579	1.66	0.00647	0.02082
hsa-miR-223	1.64	0.00775	0.02035
hsa-miR-381	1.46	0.00641	0.02104
hsa-miR-329	1.46	0.00287	0.01413
hsa-miR-769-3p	1.44	0.00861	0.01995
hsa-miR-524-3p	1.43	0.00261	0.01369
hsa-miR-93*	1.43	0.00796	0.01991
hsa-miR-220a	1.40	0.00690	0.01978
ebv-miR-BART5	1.40	0.00677	0.02015
hsa-miR-491-5p	1.39	0.00639	0.02143
hsa-miR-200c*	1.39	0.00662	0.02047
hsa-miR-133b	1.38	0.00666	0.02018
hsa-miR-19b-1*	1.37	0.00692	0.01948
hsa-miR-520h	1.37	0.00693	0.01917
hsa-miR-92b	1.35	0.00792	0.02014
hsa-miR-657	1.35	0.00801	0.01971
hsa-miR-891a	1.35	0.00818	0.01983
hsa-miR-326	1.35	0.00834	0.01991
hsa-miR-541*	1.34	0.00971	0.02126

**Table 2:** Down-regulated miRNAs in migrating glioma cells.

microRNA ID	Fold Change (Rim vs. Core)	*p*-value (T-test)	False Discovery Rate
hsa-miR-16	−8.26	0.00046	0.00564
hsa-miR-30c	−7.03	0.00043	0.00569
hsa-miR-15b	−6.93	0.00050	0.00567
hsa-let-7f	−6.58	0.00009	0.00239
hsa-let-7a	−6.46	0.00001	0.00140
hsa-miR-23b	−6.09	0.00426	0.01677
hsa-miR-103	−6.07	0.00261	0.01327
hsa-miR-107	−5.97	0.00097	0.00807
hsa-miR-24	−5.91	0.00332	0.01452
hsa-miR-93	−5.70	0.00018	0.00348
hsa-miR-15a	−5.66	0.00689	0.02011
hsa-miR-151-5p	−5.66	0.00013	0.00294
hsa-miR-23a	−5.51	0.00177	0.00995
hsa-miR-30b	−5.46	0.00051	0.00536
hsa-let-7e	−5.29	0.00002	0.00088
hsa-miR-30a	−5.29	0.00363	0.01507
hsa-miR-29a	−5.26	0.00625	0.02141
hsa-miR-455-3p	−5.14	0.00550	0.01926
hsa-let-7b	−4.90	0.00003	0.00103
hsa-miR-25	−4.79	0.00020	0.00347
hsa-let-7c	−4.74	0.00001	0.00075
hsa-miR-106b	−4.73	0.00914	0.02058
hsa-miR-92a	−4.72	0.00442	0.01700
hsa-let-7d	−4.62	0.00121	0.00831
hsa-miR-125b	−4.55	0.00493	0.01849
hsa-miR-125a-5p	−4.47	0.00134	0.00811
hsa-miR-222	−4.45	0.00520	0.01908
hsa-let-7g	−4.44	0.00113	0.00847
hsa-miR-17	−4.43	0.00331	0.01491
hsa-miR-20a	−4.27	0.00110	0.00866
hsa-miR-29c	−4.25	0.00762	0.02034
hsa-let-7i	−4.20	0.00041	0.00582
hsa-miR-26a	−4.13	0.00124	0.00780
hsa-miR-574-3p	−3.90	0.00299	0.01426
hsa-miR-181b	−3.80	0.00038	0.00606
hsa-miR-30e	−3.80	0.00066	0.00611
hsa-miR-320	−3.78	0.00064	0.00627
hsa-miR-99a	−3.51	0.00905	0.02067
hsa-miR-424	−3.45	0.00149	0.00871
hsa-miR-181a	−3.19	0.00788	0.02037
hsa-miR-10b	−3.13	0.00073	0.00638
hsa-miR-17*	−2.90	0.00234	0.01273
hsa-miR-140-5p	−2.87	0.00117	0.00836
hsa-miR-197	−2.73	0.00744	0.02022
hsa-miR-151-3p	−2.48	0.00002	0.00095
hsa-miR-30e*	−2.27	0.00123	0.00811

### Quantitative PCR analysis of miRNA expression in glioma cells

As down-regulation of miRNAs in the invasive cell population may allow for expression of genes that play important roles in glioma migration, we chose a set of candidate miRNAs down regulated in the invasive rim population with predicted gene targets pertaining to migration, invasion, and survival. We validated the results of the microarray analysis by performing quantitative PCR (qPCR) analysis for those miRNAs of interest. As in the microarray experiments, matched samples of migrating cells from the rim and migration-restricted glioma cells from the core were microdissected from the radial cell migration format. Quantitative PCR analyses demonstrate that of the 7 miRNAs examined, 5 were consistently down-regulated in the migrating cell population in all the cell lines analyzed, consistent with the microarray results (Table 3). miR-15b and miR-19 exhibited some variability being down regulated in the invasive cell population in 3/6 and 4/6 cell lines, respectively. Notably, the expression of miR-23b was significantly lower in the migratory cell population relative to the migration-restricted core cells in all six of the glioma cell lines. Among the predicted targets of miR-23b is the focal adhesion kinase Pyk2 which we have previously demonstrated plays a role in stimulating glioma cell migration [24], [25] suggesting down regulation of miR-23b may allow for extended expression of Pyk2 in the invasive cell population.

**Table 3:** qPCR analysis of differentially expressed miRNAs in migrating glioma cells.

Cell Line	Fold Change (Rim vs. Core)						
	*let-7e*	*let-7f*	*miR-15b*	*miR-16*	*miR-19*	*miR-23b*	*miR-30e*
A172	−2.200	−1.830	0.473	−2.667	0.203	−1.050	−3.120
SNB19	−4.667	−2.337	0.003	−1.543	0.373	−0.993	−3.537
T98G	−2.093	−2.047	−0.020	−.127	−1.137	−2.190	−2.853
U118	−0.587	−0.827	−1.557	−2.250	−0.620	−2.177	−0.453
U251	−2.127	−0.103	0.773	−1.183	−1.523	−2.740	−1.990
U87	−1.533	−0.120	−0.903	−2.073	−1.750	−1.160	−2.137

### Overexpression of miR-23b suppresses glioma cell migration and invasion

**Figure 3:**
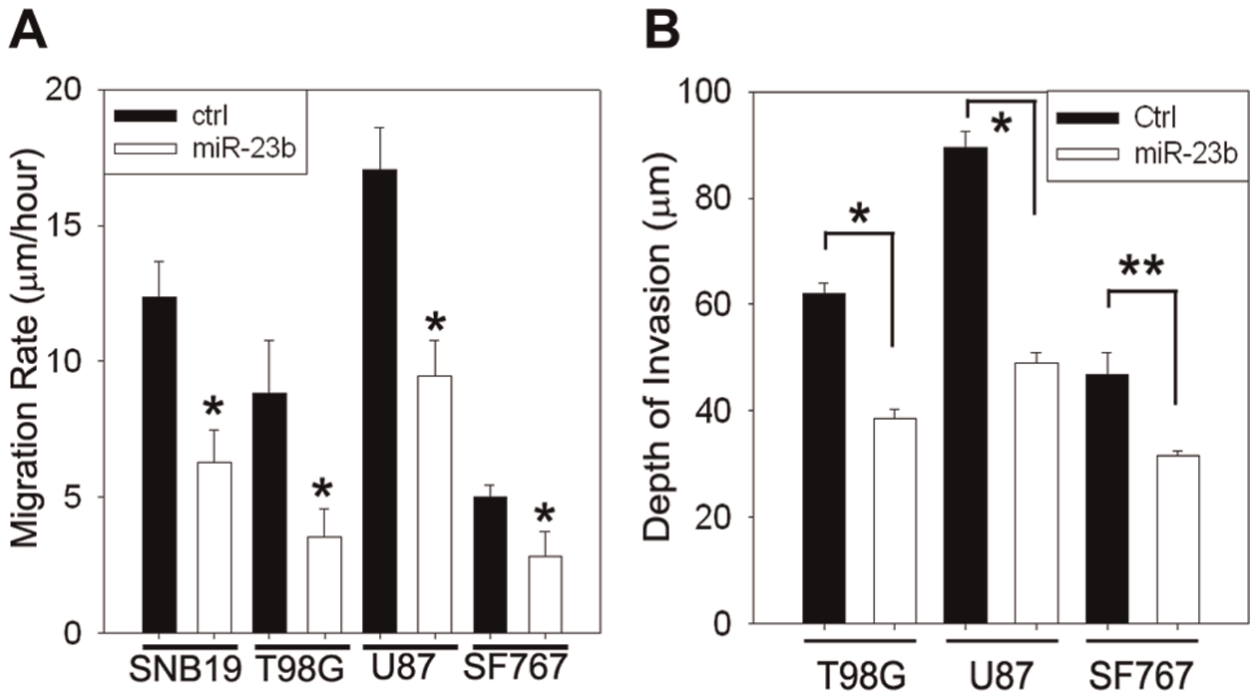
Constitutive miR-23b expression suppresses glioma cell migration and invasion. **A**. Effect of constitutive miR-23b expression on glioma migration. Glioma cells stably transduced with empty vector (ctrl) or miR23b were seeded onto 10-well glass slides pre-coated with 10 µg/ml human laminin. Cell migration was assessed over 24 hours. Data represent the average of three independent experiments and is depicted as mean ± SD (*: *p*<0.01). **B**. Effect of constitutive miR-23b expression on glioma invasion in *ex vivo* murine brain slice assay. Control cells or cells constitutively expressing miR-23b were implanted onto the bilateral putamen of mouse brain slices and observed at 48 hr. Depth of invasion was calculated from Z-axis images collected by confocal laser scanning microscopy. The mean value of the depth of invasion was obtained from six independent experiments and is depicted as mean ± SD (*: *p*<0.01, **: *p*<0.05).

To examine the effect of miR-23b expression on glioma cell migration, SF767, SNB19, T98G and U87 glioma cell lines were stably transduced with a recombinant lentivirus encoding a miR-23b precursor-microRNA. Control cells were transduced with the empty lentiviral vector alone. Stably transduced cells were enriched by flow cytometry and increased expression of miR-23b was validated by qPCR. The effect of miR-23b expression on glioma cell migration was examined using the radial cell migration assay. Overexpression of miR-23b significantly inhibited the migration rate of all four cell lines (Figure 3A). The effect of expression of miR-23b on glioma cell invasion was examined in the authentic brain matrix using an *ex vivo* organotypic brain slice model [32], [33]. Three glioma cell lines (SF767, T98G and U87) stably transduced with empty lentiviral vector or a recombinant lentivirus encoding a miR-23b precursor microRNA were seeded onto a fresh murine brain slice and allowed to invade the matrix for 48 hr. Increased expression of miR-23b significantly inhibited the invasion of all three of the cell lines relative to their matched control cells (Figure 3B) indicating increased expression of miR-23b inhibits glioma cell migration and invasion.

### Anti-miR-23b-mediated depletion of miR-23b enhances glioma cell migration

**Figure 4:**
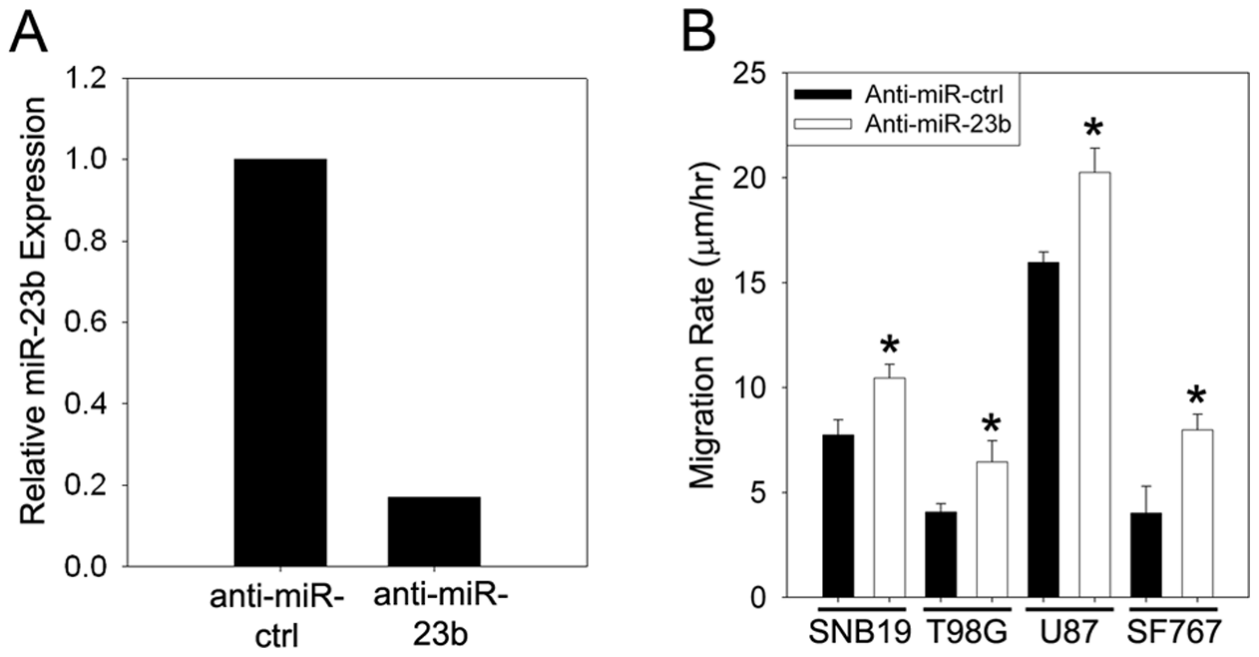
Suppression of miR-23b by anti-miR-23b oligonucleotides enhances glioma cell migration. **A.** Effect of anti-miR-23b on miR-23b expression. T98G glioma cells were transfected with 100 nM anti-miR-23b oligonucleotide or a random sequence control oligonucleotide. 48 hours after transfection, expression of miR-23b was determined by quantitative PCR. **B** pre-coated with 10 µg/ml human laminin 16 hours after transfection. Cell migration was ass**.** Effect of anti-miR-23b expression on glioma migration. Cells were seeded onto 10-well glass slides essed over 24 hr. Bars represents the average of three independent experiments (*: *p*<0.05).

To further define the role of miR-23b in the regulation of glioma cell migration, we examined the effect of knockdown of miR-23b expression in four glioma cell lines (SF767, SNB19, T98G and U87) using an anti-miR*-*23b oligonucleotide. Transfection of glioma cells with an anti-miR-23b oligonucleotide resulted in reduction of miR-23b expression by ∼ 90%. Representative results are shown for T98G cells (Figure 4A). The effect of reducing miR-23b expression on glioma cell migration was examined using the radial migration assay. The reduction of miR-23b expression resulted in a significant increase in the migration rate of all four glioma cell lines relative to cell lines expressing a control random sequence anti-miR (Figure 4B).

### Clinical samples indicate lower expression of miR-23b in the invading rim

**Figure 5:**
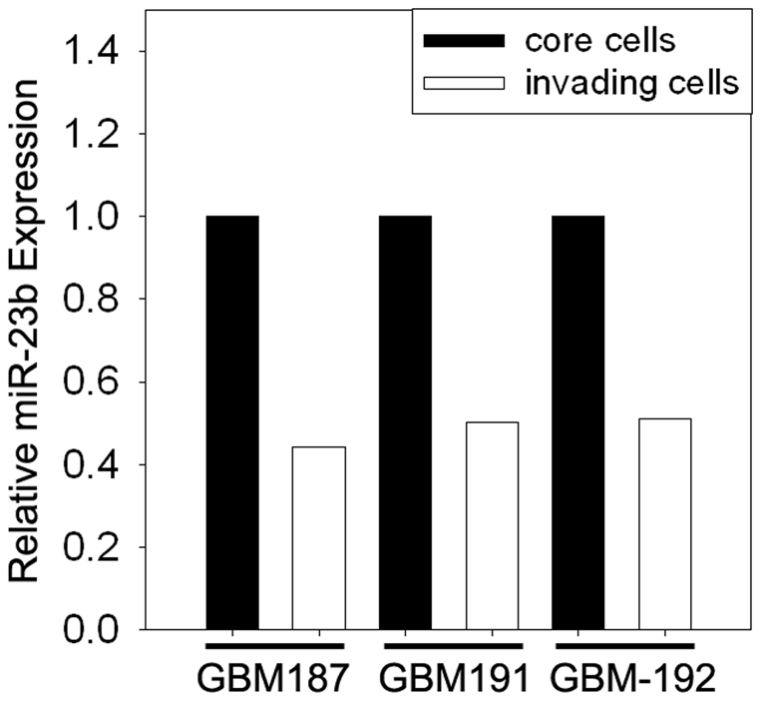
Differential expression of miR-23b in GB patient specimens. Surgical glioblastoma biopsy samples were sectioned, stained, and the tumor core or invasive rim were delineated by histological examination. Cells from the invasive rim and tumor core populations were microdissected and total RNA, including small RNA, was isolated. The relative abundance of miR-23b in each cell population was determined by quantitative PCR. Results are depicted relative to control and normalized to 18S ribosomal RNA.

Results from the *in vitro* migration assay indicate that miR-23b expression is down regulated in the migratory cell population relative to cells in the migration-restricted core. To determine whether miR-23b was differentially expressed in glioblastoma cells *in vivo*, we examined miR-23b expression in clinical biopsy samples. Cells from the invasive edge or cells from the tumor core were microdissected from tissue sections from three patient samples (GBM 187, GBM 191 and GBM 192). Quantitative PCR analysis revealed that miR-23b expression was markedly reduced in the invasive edge population compared to matched cells of the tumor core in all three patient samples (Figure 5) corroborating the results of the *in vitro* assays.

### Pyk2 is a target of miR-23b in glioma cells

**Figure 6:**
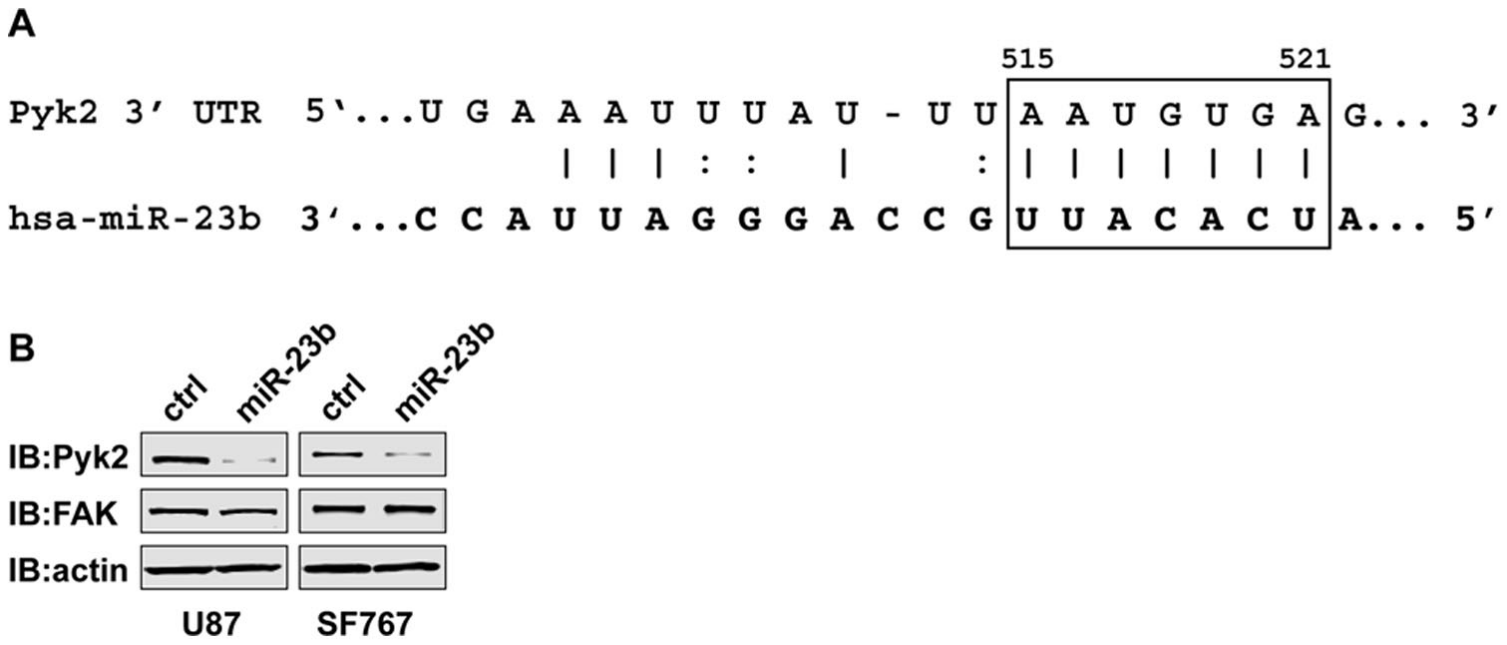
Pyk2 is a target of miR-23b in glioma cells. **A**. Predicted pairing of Pyk2 3' UTR target region (top) and hsa miR-23b (bottom). Box highlights the exact match of positions 515-522 of Pyk2 3' UTR with positions 2-8 (the seed plus position 8) of the mature miRNA. **B.** Immunoblot analyses of Pyk2 and FAK in control transduced U87 and SF767 glioma cells (ctrl) or in U87 and SF767 cells constitutively expressing miR-23b. Cell lysates were immunoblotted with anti-actin antibody to ensure equivalent protein loading in each lane.

**Figure 7:**
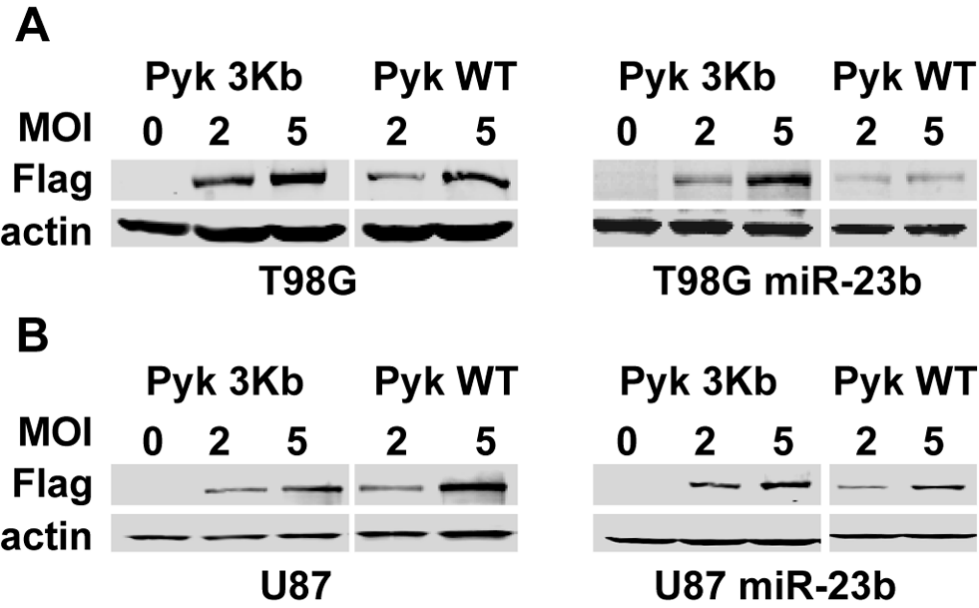
miR-23b targets the 3′ UTR of Pyk2. Wild-type T98G and U87 glioma cells or T98G and U87 cells constitutively expressing miR-23b were infected with recombinant adenoviruses expressing FLAG epitope-tagged wild-type Pyk2 (Pyk WT) or Pyk2 lacking the 3' UTR (Pyk 3Kb). Cells were infected at the indicated multiplicity of infection (MOI). 24 hr after infection, cells were lysed, and the cell lysates were immunoblotted with an anti-FLAG monoclonal antibody. Lysates were immunoblotted with anti-actin antibody to ensure equivalent protein loading in each lane.

A bioinformatics search of potential miR-23b targets using miRBase [35] and TargetScan [36], [37] revealed a conserved target site within the 3’ UTR of the non-receptor tyrosine kinase Pyk2 (Figure 6A). Increased expression and activity of Pyk2 correlates with advancing tumor grades in patients [38]. Furthermore, we have previously demonstrated that knockdown of Pyk2 expression significantly inhibited glioblastoma cell migration *in vitro* and invasion *ex vivo* in brain slices [25]. In addition, reducing Pyk2 expression levels in glioblastoma cells significantly extends survival in a murine intracranial xenograft tumor model [39]. To validate that Pyk2 expression can be regulated by miR-23b, we examined the expression of Pyk2 in miR-23b overexpressing glioma cells. Expression of Pyk2 was significantly reduced in stably transduced U87 and SF767 glioma cells overexpressing miR-23b relative to Pyk2 expression in control transduced U87 and SF767 glioma cells as assayed by immunoblotting (Figure 6B). Unlike Pyk2, the 3’ UTR of the closely related focal adhesion kinase FAK does not contain a predicted miR-23b target site [35], [36] and immunoblotting indicated that increased expression of miR-23b did not significantly alter expression of FAK in these cells. To further examine whether Pyk2 expression is modulated by miR-23b, we performed co-expression studies. Two recombinant adenoviruses were assembled for these studies. Both adenoviruses encode a FLAG epitope-tagged Pyk2 however; they differ in their respective transcripts. In the Pyk WT construct, the transcript includes the entire coding sequence and 725 bp of 3' UTR that includes the potential miR-23b target sequence. In the Pyk 3Kb construct, the transcript is limited to the coding sequence and lacks any 3' UTR. Infection of parental T98G or U87 glioma cells with matched MOI of either the Pyk2 WT or Pyk 3Kb recombinant adenoviruses resulted in dose dependent expression of Pyk2 protein as assayed by immunoblotting (Figure 7). Infection of T98G or U87 cells overexpressing miR-23b with the Pyk 3Kb adenovirus produced dose-dependent expression of Pyk2 protein similar to that observed in the parental cells. In contrast, expression of Pyk2 protein in the T98G or U87 cells overexpressing miR-23b following infection with the Pyk WT adenovirus was significantly reduced relative to Pyk2 expression in the parental cells at the same MOI, substantiating that Pyk2 is a target for miR-23b (Figure 7).

**Figure 8:**
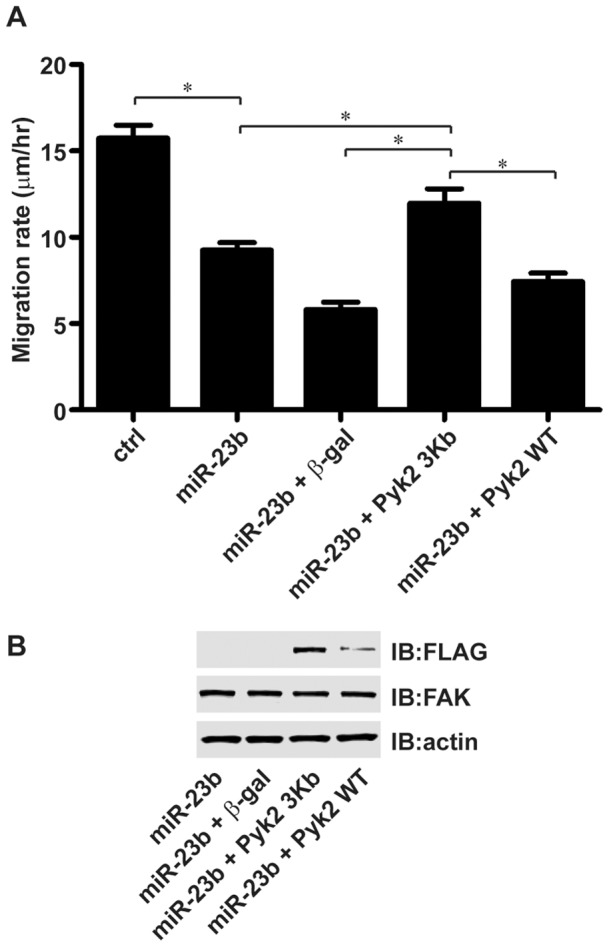
Re-expression of Pyk2 stimulates migration of miR-23b overexpressing cells. **A**. T98G cells transduced with empty vector (ctrl) were left uninfected while T98G cells overexpressing miR-23b were infected with adenoviruses encoding ß-galactosidase, or FLAG epitope-tagged wild-type Pyk2 (Pyk WT) or Pyk2 lacking the 3' UTR (Pyk 3Kb) at MOI = 5. Sixteen hours after infection, cells were plated onto laminin coated slides and the migration rate determined after 24 hours. Values are mean ± SD of 10 replicate samples (*: *p*<0.05). **B**. Whole cell lysates were immunoblotted with the indicated antibodies.

To determine the functional relationship between Pyk2 and miR-23b expression, we examined the capacity of increased Pyk2 expression to overcome the inhibition of glioma cell migration mediated by miR-23b in the radial migration assay (Figure 8). Consistent with earlier results, the migratory rate of T98G cells with increased miR-23b expression was significantly reduced compared to control transduced T98G cells. Infection of T98G miR-23b cells with the Pyk 3Kb recombinant adenovirus significantly increased their migration rate relative to T98G miR-23b cells and T98G miR-23b cells infected with a control adenovirus encoding ß-galactosidase. Increased migratory rate was accompanied by increased expression of Pyk2 as assayed by immunoblotting. In contrast, infection of T989G miR-23b cells with a matched MOI of the Pyk WT recombinant adenovirus resulted in only a modest increase in Pyk2 expression and did not significantly increase the migratory rate relative to the T98G miR-23b cells. Immunoblotting also indicated that FAK expression was equivalent in each of the experimental cells indicating that FAK was unable to fully compensate for the reduction in Pyk2 expression consistent with previous results [25]. Together these results indicate Pyk2 is a target for miR-23b and that miR-23b modulates glioma cell migration, in part, by regulating Pyk2 expression.

## Discussion

It is now well appreciated that miRNAs play a central role in a broad range of physiological processes by dynamically regulating gene expression. We have been interested in examining alterations in gene expression profiles of invasive glioma cells to identify potential targets to limit glioma cell invasion, increase their susceptibility to cytotoxic agents, and improve clinical outcomes in GB. In the current study, we investigated the potential role of differential expression of miRNAs in glioma invasion by comparing the matched large-scale, genome-wide miRNA expression profiles of migrating and migration-restricted human glioma cells. The major findings of this report are as follows: (1) a conserved set of miRNAs was identified that was consistently down-regulated in the migrating cell population relative to cells in the migration-restricted population, (2) miR-23b was significantly down regulated in migrating glioma cells *in vitro* and in cells from the invasive edge in patient tumor samples, (3) overexpression of miR-23b significantly inhibited glioma cell migration and invasion while inhibition of miR-23b expression significantly increased glioma cell migration, (4) predictive miRNA target analysis revealed a conserved miR-23b target site within the 3’ UTR of Pyk2, a non-receptor tyrosine kinase previously implicated in the regulation of glioma cell migration and invasion, (5) increased expression of miR-23b reduced the expression level of Pyk2 in glioma cells but did not significantly alter the expression level of the related focal adhesion kinase FAK, (6) expression of a Pyk2 transcript, devoid of the 3’UTR containing the miR23-b target sequence, in miR-23b over-expressing cells increased Pyk2 protein expression and partially rescued glioma cell migration. Together, these data indicate reduced expression of miR-23b, in part, facilitates glioma cell migration and invasion via modulation of Pyk2 expression.

The propensity of malignant glioma cells to invade into the surrounding normal brain parenchyma precludes effective clinical treatment. The inability to effectively target the invasive cells virtually assures tumor recurrence. Therefore, understanding the molecular determinants of glioma that regulate invasion is essential for the development of new therapies and improved clinical outcomes. While recent studies have identified a role for miRNAs in GB [17], [18], [22], [40], a detailed analysis of global miRNA expression profiles in matched samples of migrating or migration-restricted glioma cells has not been previously reported. Our analysis of a panel of glioma cell lines identified a set of miRNAs that were consistently down regulated in the migratory cell population, suggesting a potential role for these miRNAs in the regulation of migration. miR-23b was included in the set of down-regulated miRNAs and predictive miRNA target analysis revealed several known drivers of cell migration and invasion as potential targets of miR-23b. Indeed, increased expression of miR-23b significantly inhibited the migration rate of glioma cells *in vitro* and invasion *ex vivo* in brain slices. Conversely, suppression of miR-23b expression stimulated the migration rate of glioma cells. These results were substantiated by the demonstration that miR-23b expression was significantly reduced in glioma cells from the invasive rim in patient GB samples. Similarly, recent reports have demonstrated that miR-23b expression is significantly down regulated in several cancers including metastatic prostate, hepatocellular, bladder, and colon cancer [41], [42], [43], [44]. Our data indicate that alterations in miR-23b expression may also play a role in GB invasion and progression.

Expression of miR-23b has recently been demonstrated to regulate the expression of a set of pro-metastatic genes [44]. Our analyses indicate that the non-receptor focal adhesion kinase Pyk2 also represents a potential target for miR-23b. Several lines of evidence support a role for Pyk2 in glioma invasion. Expression of Pyk2 increases with increasing tumor grade in patient GB samples [38]. In addition, our previous studies have demonstrated that the migration rate of glioma cells *in vitro* correlated positively with Pyk2 activity [24]. Increased expression of Pyk2 increased glioma cell migration and expression of Pyk2 is upregulated in invasive glioma cells *in situ* relative to the cells in the tumor core [11], [24], [25]. Conversely, silencing of Pyk2 expression significantly inhibited glioma cell migration *in vitro*, inhibited glioma cell invasion in brain slices, and prolonged survival of intracranial xenografts [25], [39]. Our current data indicate that miR-23b regulates Pyk2 protein expression. Increased expression of miR-23b suppressed Pyk2 expression which correlated with a reduced glioma cell migration rate *in vitro* and invasion *ex vivo* in brain slices. In addition, the observed reduction of miR-23b expression in the invasive cell population of human GBM biopsy samples is consistent with the increased expression of Pyk2 observed in the invasive cell population [11] and support a potential mechanism for the maintenance of an invasive phenotype via modulation of Pyk2 expression. Future studies will seek to investigate the effect of altering miR-23b expression on glioma cell invasion with primary GBM xenografts [45] in orthotopic mouse models.

Cellular invasion is a dynamic process dependent upon the interplay among diverse signaling pathways initiated by cues from the cellular and extracellular environments [46], [47]. Alterations in miRNA expression can directly as well as indirectly regulate signaling pathways by modulating the expression of important signaling effectors. Our findings indicate a role for miR-23b regulation of Pyk2 expression in glioma migration however, given the capacity of miRNAs to bind to targets through imperfect matching it is expected that downregulation of miR-23b alters the expression of multiple proteins in addition to Pyk2. Indeed, bioinformatic analysis suggests that miR-23b can potentially regulate the expression of over 100 genes and a recent study has validated miR-23b targeting of a set of genes implicated in invasion, metastasis, and angiogenesis [44]. The identity of other miR-23b potential targets related to glioma migration and their relationship to Pyk2 expression and signaling remains to be determined. In this regard, it is noted that increased expression of Pyk2 utilizing a non-targetable Pyk2 transcript in miR-23b overexpressing cells was capable of stimulating cell migration but did not completely rescue the migration deficit substantiating a requirement for other miR-23b targets in glioma migration. Moreover, since our analysis indicated that a set of miRNAs was down regulated in the migratory cell population, it is likely that targets of other miRNAs in this set, in addition to miR-23b targets, also participate in the complex regulation of glioma invasion and warrant further investigation.

In summary, the results of the current study assign an important role for miR-23b expression in the regulation of glioma cell migration, in part, through modulation of Pyk2 expression. Additional studies will be required to identify other targets of miR-23b and their role in the regulation of glioma cell migration and invasion. Given the critical need for therapies that effectively target the invasive cell component of glioma, these data suggest that selective modulation of specific miRNAs may provide a rich area for therapeutic development to improve clinical outcome in GB.
